# The Lethal Effect of a Nano Emulsion of *Satureja hortensis* Essential Oil on Protoscoleces and Germinal Layer of Hydatid Cysts

**Published:** 2019

**Authors:** Mohammad MOAZENI, Mohammad Jamal SAHARKHIZ, Amir Mootabi ALAVI

**Affiliations:** 1. Department of Pathobiology, School of Veterinary Medicine, Shiraz University, Shiraz, Iran; 2. Department of Horticultural Science, School of Agriculture, Shiraz University, Shiraz, Iran

**Keywords:** Hydatid disease, Treatment, Scolicidal, *Satureja hortensis*, Nano emulsion

## Abstract

**Background:**

New scolicidal agents and novel therapeutic drugs are essential for better management of the zoonotic infection, hydatid disease. This study evaluated the effect of a nanoemulsion (NE) of *Satureja hortensis* essential oil (SHEO) on protoscoleces and germinal layer of hydatid cysts.

**Methods:**

This study was conducted from July to October 2016 in Shiraz University, Shiraz, Iran. Gas chromatography (GC) and gas chromatography-mass spectrometry (GC-MS) were performed to identify the main components of SHEO. To determine the scolicidal power of the NE of SHEO, live protoscoleces of hydatid cysts were exposed to two concentrations (1 and, 2 mg/mL) of the NE and incubated at 37 °C for 10 and 20 min. To evaluate the anti-hydatid effect of the NE of SHEO, the collected hydatid cysts from the abdominal cavities of the experimentally infected mice were immersed in the NE (0.5 mg/ml) and incubated at 22 °C for 24 h.

**Results:**

Carvacrol and γ-terpinene were the major components of the SHEO. NE of SHEO at the concentrations of 1 and 2 mg/mL showed 100% scolicidal power after 20 and 10 min respectively. Exposure of the hydatid cysts to the NE of SHEO resulted in crumpling of their germinal layer and detachment of this layer from the laminated layer.

**Conclusion:**

NE of SHEO showed a strong scolicidal activity as well as a profound lethal effect on the germinal layer of hydatid cysts. Accordingly, this product may be used as a natural scolicidal agent in hydatid cyst surgery. Furthermore, it may be used as a therapeutic tool for treatment of hydatid disease.

## Introduction

**H**ydatid cyst, the larval stage of *Echinococcus granulosus* is an important zoonotic infection in many countries of the world. The final hosts including dogs and some other carnivores, release the infective eggs in their feces and spread the infection between intermediate host including humans and domestic livestock such as cattle, sheep, goats, camels, horses, pigs, and others. After ingestion of infective eggs by the intermediate hosts, hexacanth embryos release from the eggs, penetrate the intestinal mucosa, enter the systemic circulation, reach different organs and grow there to form the hydatid cysts after several months (1, 2).

Each hydatid cyst consists of an acellular laminar layer and a germinal layer that produces brood capsules containing several protoscoleces (3). Hydatid cysts are mainly formed in the liver (50%–70% of all cysts), lung (20%–30%) and less frequently in other organs (4).

In surgery as the most common method of treatment, there is the danger of intraoperative leakage of scolices, which may cause the recurrence of new hydatid cysts. Inactivation of cyst contents before surgery, using an effective scolicidal agent, may considerably decrease the risk of recurrence (5). Various scolicidal agents including cetramide (6), 10% povidone-iodine (7), 20% Silver nitrate (8), H_2_O_2_ (9), 20% hypertonic saline (8) and 95% ethyl alcohol (9) had high scolicidal activity. On the other hand, use of most of these agents is accompanied by adverse side effects and undesirable complications (5, 10–12). Therefore, there is still no ideal effective and safe scolicidal agent. This is why surgeons need safer and more efficient scolicidal agents for use in surgery of hydatid cysts (13).

In addition, further in vitro and in vivo investigations should be undertaken to find more effective and safer therapeutic drugs for treatment of hydatid disease (14).

*Satureja hortensis* L. (summer savory), belonging to the Lamiaceae family, is a well-known medicinal plant and is widely distributed in Turkey and southwest and central Asia including Iran. The aerial parts of *S. hortensis* are often used as tea or additive in condiments for various foods (15). The essential oil of *S. hortensis* had antimicrobial (16). Antibacterial (17), antioxidant (17) and antifungal (18) properties.

Since *S. hortensis has* a number of medicinal properties, the present study was designed to evaluate the scolicidal power of the nano emulsion (NE) of *Satureja hortensis* essential oil (SHEO) and its influence on the germinal layer of hydatid cysts.

## Materials and Methods

### Collection of Plant material

This study was conducted from July to October 2016 in Shiraz University, Shiraz, Iran. At the beginning of the fruit set stage, *S. hortensis* aerial parts were harvested from Experimental field of Agriculture College, Shiraz University, Shiraz, Iran. The experimental field is situated at 1810 m above the sea level, altitude 52º 32′ east and latitude 29º 36´ north having a semi-arid climate. Average maximum and minimum temperatures of the field in current 10-yr was 38 °C and − 10 °C respectively. Identification and authentication of the plant species were done by a plant taxonomist of Shiraz University (Prof. A. R. Khosravi) and a voucher specimen was kept in the herbarium.

### Preparation of SHEO

The samples were firstly dried in shade at room temperature (less than 25 °C) for 14 d. Then 30 g of the powdered sample was hydrodistillated for 3 h, by an all-glass Clevenger-type apparatus with regard to the process characterized by the European Pharmacopoeia (1983) (19). After separation from the aqueous layer, the EO was collected and dried over anhydrous sodium sulfate. The obtained EO was stored in sealed vials at 4 °C till use. Approximately 20 g of EO was obtained from 1000 g of dried powder of *S. hortensis.*

### Preparation of NE of SHEO

Nanoemulsion of SHEO was prepared through low energy system using 96% (v/v) water, 2% (v/v) EO and 2% (v/v) Tween 80. The EO and Tween 80 were stirred at 2400rpm using a homogenizer for 20 min (20). Afterward, water was added gently to the mixture. The obtained mixture was further stirred at 3000rpm for 30 min. The resulting NE was kept at 4 °C until use. Almost 1000 ml of NE of SHEO at concentration of 10mg/ml. was obtained from 10 g of EO.

### NE droplet size determination

Emulsion droplet size was measured by dynamic light scattering (DLS) using NANO-flex® 180° (USA). To avoid multiple scattering effects, the emulsion samples were diluted to 10% with deionized water before size determination. Emulsion droplet size was estimated by the average of three measurements (21). The mean droplet size was 90 nm.

### Essential oil analysis procedure

The ingredients of volatile oil from the aerial parts of plant were identified using GC and GC-MS analyses (22). The GC analysis was done by an Agilent gas chromatograph series 7890-A having a FID (flame ionization detector). Analysis was performed on fused silica capillary HP-5 column (30m × 0.32mm i.d.; film thickness 0.25μm). The sample volume injected into the GC was 0.2 μL pure EO. The temperature of injector and detector was set at 250 °C and 280 °C, respectively. Nitrogen acted as carrier gas with a flow rate of 1ml.min; the oven temperature was 60%–210°C with the rate of 4 °C/min, which was then increased to 240 °C with the rate of 20 °C min, and finally, held isothermally for 8.5 min. The split ratio was 1:50. The GC–MS analysis was accomplished using an Agilent gas chromatograph equipped with fused silica capillary HP-5MS column (30m × 0.25mm i.d.; film thickness 0.25μm) coupled with 5975-C mass spectrometer. 0.1μL pure EO with the split ratio of 1:50 was injected into the capillary column. Helium with the ionization voltage of 70 eV acted as carrier gas. The temperature of interface and ion source was 280 °C and 230 °C, respectively. Mass range was from 45 to 550amu. The oven temperature program was the same as for the GC. The retention indices for all components were specified using n-alkanes as standard.

### Identification of essential oil components

The compounds were recognized by comparing their retention indices (RI, HP-5) with the reported ones in the literature and by comparing their mass spectra with the Adams Library, Mass Finder 2.1 Library and Wiley GC–MS Library data as well as the published mass spectra data (23).

### Collection of protoscoleces

Live protoscoleces of *E. granulosus* were collected aseptically from the hydatid cysts obtained from the liver of naturally infected sheep slaughtered in Shiraz (South of Iran). The fluid of hydatid cysts was aspirated and transferred to glass containers and left unmoved for 30 min. The protoscoleces gathered at the bottom of the dishes. Then the upper hydatid fluid was removed, and protoscoleces were washed with sterile normal saline. The viability of protoscoleces was confirmed by their activity under light microscope (×40–×100) and 0.1% eosin staining. The live protoscoleces were placed in dark containers containing normal saline and stored at 4 °C until use.

### In vitro scolicidal tests

**Fig. 1:**
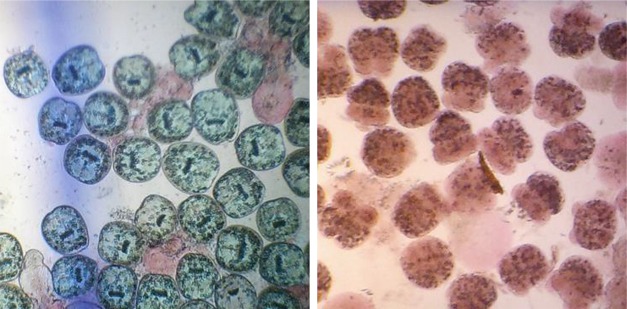
Protoscoleces of *Echinococcus granulosus* after staining with 0.1% eosin, right: exposured to nanoemulsion of *Satureja hortensis* essential oil, left: control group. Note the stained and destructed protoscoleces in the right photograph

In the first step of this study, protoscoleces were added to two concentrations of the NE of SHEO solution (1 and, 2 mg/mL) for 10 and 20 min. Since the concentration of the stock solution of NE of SHEO was 10mg/ml, to prepare the above concentrations, 1 and 2mL of the stock solution were added to 9 and 8 mL of normal saline, in two test tubes, respectively. For each experiment, 2 mL of the obtained solution was transferred into a small test tube, subsequently one drop of the sediment containing 1100–1500 protoscoleces was added to the tube using a Pasteur pipette. After gently mixing the contents, the tube was incubated at 37 °C. At the end of incubation times (10 and 20 min), the upper part of solution was carefully discarded and one ml of 0.1% eosin stain (1 g of eosin powder in 1000 mL distilled water) was added to the specimens and mixed gently. After 15 min of incubation, the upper fluid was removed carefully and the remaining protoscoleces were placed on a scaled glass slide. After covering with a coverslip the specimens were examined using a light microscope. The scolicidal activity was determined by counting a minimum of 1100 protoscoleces. The protoscoleces in the control group were exposed only to normal saline. Protoscoleces with absorbed dye were considered as dead, otherwise were recorded as potentially viable (24) (Fig. 1). All experiments were done in triplicate.

### Evaluation of the anti-hydatid effect of NE of SHEO

Five laboratory mice were experimentally infected with 1500 viable protoscoleces. After development of hydatid cysts (6 months after infection), the infected mice were euthanized, necropsied and the cysts were carefully recovered and collected in Petri dishes containing normal saline. Then the cysts with at least 5 mm diameter were selected for the experiments. After several washing in normal saline, the selected cysts were divided into test and control groups (12 in each). The cysts of the test group were placed in a small glass container with screw cap containing 15 ml of NE of SHEO at concentration of 0.5 mg/ml. while the cyst of the control group was placed in the same dish containing 15 ml normal saline. The dishes of the test and control groups were incubated at room temperature (∼ 22 °C) for 24 h.

### Ethics

All experiments were carried out in accordance with the guidelines for the care and use of laboratory animals established by the Ethics Committee of Shiraz University and unnecessary animal suffering was avoided throughout the study.

## Results

The GC and GC–MS analyses of the EO revealed the presence of 30 components constituting 99.89% of the total oil (Table 1). Carvacrol and γ-terpinene were the main oil components of *S. hortensis* EO, representing 55.6% and 31.9% of the total, respectively. The scolicidal effects of NE of SHEO at concentrations of 1 and, 2 mg/mL and for two exposure times (10 and 20 min) are presented in Tables 2 and 3 respectively. While the death rate of protoscoleces in the control group was 4.46%, when protoscoleces were exposed to NE of SHEO at a concentration of 1 mg/mL, the death rate increase to 88.40% and 100% after 10 and 20 min, respectively. NE of SHEO at a concentration of 2 mg/mL showed 100% scolicidal power after 10 min. The NE of SHEO has high in vitro scolicidal activity on the protoscoleces of hydatid cysts (Fig. 1) and this effect was both dose and time-dependent.

**Table 1:** Chemical composition (%) of *Satureja hortensis* essential oil identified by Gas chromatography (GC) and gas chromatography-mass spectrometry (GC-MS)

***Number***	***Component***	R I*	***(%)***
1	α- Thujene	924	1.15
2	α-Pinene	932	0.64
3	Camphene	946	0.06
4	Hepten-1-ol	958	0.05
5	Sabinene	969	0.01
6	β-Pinene	974	0.21
7	3- Myrcene	988	1.15
8	Phellandrene	1002	0.23
9	α-Terpinene	1014	3.75
10	p-Cymene	1020	2.19
11	Sylvestrene	1025	0.37
12	E-β- Ocimene	1044	0.07
13	γ-Terpinene	1054	31.98
14	Terpinolene	1086	0.05
15	trans-α Sabinene hydrate	1098	0.07
16	Isoborneol	1155	0.06
17	Terpinene-4-ol	1174	0.2
18	α-Terpineol	1186	0.1
19	carvacrol methyl ether	1241	0.09
20	Thymol	1289	0.8
21	Carvacrol	1298	55.66
22	Thymol acetate	1349	0.03
23	Carvacrol acetate	1370	0.07
24	Caryophyllene	1417	0.36
25	Aromadendrene	1439	0.08
26	α –Humulene	1454	0.01
27	Bicyclogermacrene	1500	0.18
28	Bisabolene	1505	0.21
29	Unknown	-	0.01
30	Spathulenol	1577	0.04

* RI = Retention indices

**Fig. 2:**
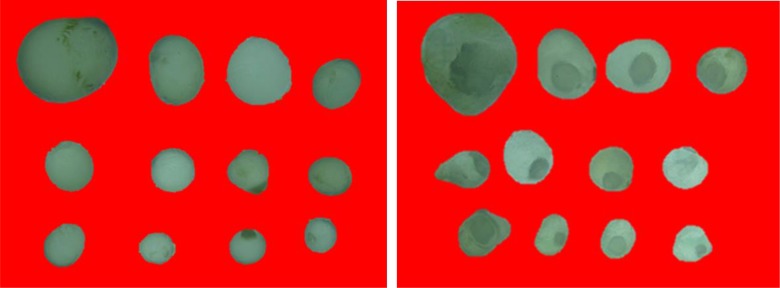
The effect of nanoemulsion of *Satureja hortensis* essential oil (0.5 mg/ml) on the germinal layer of hydatid cysts after 24h of incubation at room temperature. Note the normal germinal layer in the cysts incubated in normal saline(left) and detached and crumpled germinal layer in the cysts incubated in nanoemulsion of *Satureja hortensis* essential oil (right)

Lethal effect of NE of SHEO on hydatid cysts obtained from the experimentally infected laboratory mice are shown in Fig. 2. Macroscopic examination showed the destructive effect of NE of SHEO at a concentration of 0.5 mg/mL on the germinal layer of hydatid cysts. While the cyst structure and the germinal layer of those incubated in normal saline were normal in appearance, the germinal layer of the cysts incubated in NE of SHEO were completely detached from the laminated layer and had a crumpled appearance.

## Discussion

In the present study, the NE of SHEO was used to inactivate the protoscoleces of hydatid cysts in a laboratory trial. Numerous chemical agents have been used as scolicides against hydatid cysts, but most of them are accompanied by harmful side effects (5, 10–12).

We obtained 100% in vitro scolicidal activity using the NE of SHEO at a concentration of 1 and 2 mg/ml after 20 and 10 min of exposure respectively (Tables 2 and 3).

**Table 2:** Scolicidal effect of the naon emulsion of *Satureja hortensis* essential oil at concentration of 1 mg/ml after 10 and 20 min of application

***Exposure time (min)***	***Experiments***				
		1	2	3	Total
10	Protoscoleces	1338	1482	1319	4139
	Dead protoscoleces	1183	1304	1172	3659
	Mortality rate (%)	88.42	87.99	88.86	88.40
20	Protoscoleces	1358	1339	1412	4109
	Dead protoscoleces	1358	1339	1412	4109
	Mortality rate (%)	100	100	100	100
Control	Protoscoleces	1191	1270	1567	4028
	Dead protoscoleces	73	68	39	180
	Mortality rate (%)	6.13	5.36	2.56	4.46

**Table 3:** Scolicidal effect of the naon emulsion of *Satureja hortensis* essential oil at concentration of 2 mg/ml after 10 minutes of application

	***Experiments***	***Protoscoleces***	***Dead protoscoleces***	***Mortality rate (%)***
Test	1	1411	1411	100
	2	1397	1397	100
	3	1273	1273	100
	Total	4081	4081	100
Control	1	1191	73	6.13
	2	1270	68	5.36
	3	1567	39	2.56
	Total	4028	180	4.46

Best scolicidal agents are those that are nontoxic and destroy the scolices with a low concentration and during a short time. With regard to the outcomes of the present study, the scolicidal power of NE of SHEO at a concentration of 1 mg/mL (20 min) or 2 mg/mL (10 min) was similar to scolicidal effect of 1.5% cetrimide (10 min), 10% povidone iodine (15 min), 20% silver nitrate (20 min), 3% H_2_O_2_ (15 min), 20% hypertonic saline (45 min), and 95% ethyl alcohol (15 min). On the other hand, we obtained 100% scolicidal activity with an herbal product at lower concentration and in shorter exposure time in comparison with chemical scolicidal agents.

Now, only albendazole and mebendazole are approved for treatment of human echinococcosis. Inevitably, novel, more suitable and more effective therapeutic agents are required to optimize the treatment of hydatid disease (25). Embryonic cells existing on germinal layer of hydatid cyst can develop new protoscoleces or brood capsules (26). Accordingly, to destroy the hydatid cyst, this layer should be demolished (27). In the present study, we obtained a profound lethal effect on the germinal layer of hydatid cysts following the in vitro application of NE of SHEO (Fig. 2).

Like our study, previous studies also have shown that carvacrol and γ-terpinene are among the main compounds of *S. hortensis* essential oil (17). Carvacrol as a lipophilic compound; could simply enter into the cell membrane, damaging its integrity, changing its permeability and releasing the cellular contents. It also may interact with the enzymes involving in the synthesis of cell membrane (28). On the other hand, the in vitro and in vivo activity of carvacrol against the larval stage of *E. granulosus* have been recently documented (29). With regard to these issues, the scolicidal and anti-hydatid activity of *S. hortensis* can be explained by the presence of the phenolic compounds such as carvacrol in the essential oil of this medical plant. Carvacrol as the main ingredient of SHEO is a harmless compound and it can be considered as a safe food additive (29). The LD50 of carvacrol in mice was recently found to be 919 mg/kg after oral administration (30).

In this study, we prepared and used the NE of SHEO. Nano emulsions may reach the target organ more easily due to their small size. Long-time stability, higher water solubility and higher ability to penetrate through the biological membranes are the other privileges of nonoemulsions (31). The results of the present study revealed that the NE of SHEO is not only a powerful scolicidal agent, but also it may be considered as a good natural source for production of new anti-hydatid drugs.

## Conclusion

NE of SHEO showed a strong scolicidal power as well as a profound destructive effect on the germinal layer of hydatid cysts following the in vitro application of this herbal product. With regard to the results of this study, NE of SHEO may be used as a natural scolicidal agent in hydatid cyst surgery. Furthermore, NE of SHEO either alone or along with albendazole, may be used as a therapeutic tool for treatment of hydatid disease. However, further experimental studies are required to confirm the safety of NE of SHEO in laboratory animals before recommendation of this herbal medicine for treatment of hydatid disease in human beings.
